# A review on pathogenicity of *Aeromonas hydrophila* and their mitigation through medicinal herbs in aquaculture

**DOI:** 10.1016/j.heliyon.2023.e14088

**Published:** 2023-02-28

**Authors:** Anurag Semwal, Avdhesh Kumar, Neelesh Kumar

**Affiliations:** Department of Aquaculture, College of Fisheries, Govind Ballabh Pant University of Agriculture and Technology (GBPUA&T), Pantnagar, Uttarakhand, 263145, India

**Keywords:** *Aeromonas hydrophila*, Motile Aeromonas Septicemia (MAS), Immunostimulants, Phytotherapy, Herbalism, Pathogenicity, Hemorrhagic septicemia

## Abstract

*Aeromonas hydrophila* is a freshwater, facultatively anaerobic, chemo-organoheterotrophic bacterium that distressed fishes with gastroenteritis, septicemia and causes a disease known as Motile Aeromonas Septicemia (MAS), which affects the aquatic environment. Haemolysin, aerolysin, cytosine, gelatinase, enterotoxin and antimicrobial peptides have been identified as virulence factors in *A. hydrophila*. Medicinal herbs/plants and their uses are the instant, easily available, cost-effective, efficient and eco-friendly approach for socio-economic, sustainable development of modern aquaculture practice. Phytotherapy either through a dip or by incorporation into the diets is an alternative approach to synthetic pharmaceuticals to diminish the pathogenicity of aquatic environmental pathogens. Due to the presence of remarkable phytoconstituents like flavonoids, alkaloids, pigments, terpenoids, steroids and essential oils, the medicinal plant exhibits anti-microbial, appetite-stimulating, anti-stress, growth-promoting and immunostimulatory activities. Aqua-industry preferred phytotherapy-based techniques/compounds to develop resistance against a variety of aquatic pathogens in culturable fishes because they are inexpensive and environment-friendly. As a result, this review elaborates on the diverse applications of phytotherapy as a promising tool for disease management in aquaculture and a major step toward organic aquaculture.

## Highlights

• *Aeromonas* spp. causes significant economic losses to aquaculture.
• Medicinal plants are biocompatible and cost-efficient.
• Potent bioactive compounds of medicinal plants exhibit antimicrobial activities.
• Phytotherapy represents a promising tool for the sustainability of the aquaculture.

## 1 Introduction

*Aeromonas hydrophila* is a freshwater, facultatively anaerobic, chemo-organoheterotrophic bacterium that causes disease in fishes, amphibians, reptiles, birds and mammals with gastroenteritis, septicemia and necrotizing fasciitis being the most prevalent kinds of disease [[1], [2], [3], [4]]. *Aeromonas* species can be found in a variety of aquatic and environmental habitats including sediment, estuaries, seaweed, sea grass, used water, drinking water and food [5,6]. Genus *Aeromonas* comprises Gram-negative, motile bacilli or coccobacilli rods, non-spore-forming with rounded ends that size 1–3.5 μm across and belongs to the *Aeromonadaceae* family of *Gammaproteobacteria.* They are facultatively anaerobic, catalase, oxidase and indol-positive, able to convert nitrate to nitrite and are generally resistant to the vibrio static agent O/129. In a microbiological survey, *A. hydrophila* is prevalent in the Chesapeake Bay and its tributaries with concentrations ranging from ca. 4.6 × 10^2^/g in sediment and <0.3/l to 5 × 10^3^/ml in the water column [7]. Kaper et al. [8] found that *A. hydrophila* in shellfish growing waters had cell counts ranging from 3 to 2400 cells/100 ml in water and from 3 to 4600 cells/100 g in oysters.

Carps are the major group of freshwater fish that are important as food sources and study models all around the world. *Aeromonas* sp. and *Pseudomonas* sp. are the most prevalent bacteria isolated from carp culture systems [9]. *A. hydrophila* is a widely investigated bacteria due to its occurrence in the estuaries [10], food [11], water [12], antibiotic resistance and potential to cause disease in animals and humans [13]. Recent research found motile species of *Aeromonas* especially *A. hydrophila* are the main causing agents for a variety of infections [14]. Aeromoniasis was shown to be the most prevalent bacterial disease occurring whole year in Indian major carps *Catla catla, Labeo rohita, Cirrhinus mrigala* and exotic carps such as *Hypophthalmichthys molitrix, Ctenopharyngodon idella* and *Cyprinus carpio. H. molitrix* was the most sensitive to *Aeromonas* of the six fish species tested [9]. *A. hydrophila* has a natural habitat in water and can thrive at temperatures ranging from 0 to 45 °C with an optimum temperature of 22–32 °C. In fish, *A. hydrophila* infection is a zoonotic disease, i.e., it may be transmitted from animals to humans and vice versa [15]. Stress conditions such as crowding, low dissolved oxygen, higher organic content, physical injuries, temperature fluctuation and factory pollution may cause *A. hydrophila* infection [16,17].

*A. hydrophila* is classified as a primary or secondary pathogen [18,19]. When a pathogen causes disease in stressed fish on its own, it is referred to as a primary pathogen. Generally, *A. hydrophila* is found as a secondary invader [20]. Because secondary pathogens have a limited invasive capacity, they depend on the existence of primary infection to infect. *A. hydrophila* is usually considered a secondary pathogen that infects a fish that has already been infected with another infection [21]. *A. hydrophila* can also act as an opportunistic invader, infecting fish under stressed conditions or along with other pathogens [22]. It is considered an efficient biomarker of a stressed or polluted aquatic environment [23]. The term “opportunistic pathogen” means, if a chance is given, *A. hydrophila* always has the potential of causing disease [20].

In India, “Mrgayurveda” a subdiscipline of Ayurveda, focuses on animal life and the use of herbal medicines to treat animal diseases [24]. Phytotherapy is a medical practice that focuses more on traditional approaches rather than modern medication. It highly involves the knowledge and usage of medical herbalism. Although the aquaculture industry has only just begun using phytotherapy, it is gradually being recognized as a treatment option in place of synthetic pharmaceuticals [25]. This biodegradable and environmental-friendly application is known as phytotherapy or more often commonly called herbalism. Globally, the use of medicinal herbs in aquaculture has drawn considerable interest and has become a subject of active scientific research [24,26]. It has been observed that medicinal plants contain a wide range of appetite-stimulating, growth-promoting, antibacterial, immunostimulant, anti-inflammatory, antistress, anticancer qualities and their usage in traditional medicine has been recognized across the world for thousands of years. The most common medicinal plants incorporated in fish diets as powder and extracts are *Azadirachta indica, Withania somnifera*, *Allium sativum*, *Zingiber officinale*, *Ocimum sanctum*, *Tinospora cordifolia*, *Aloe barbadensis* etc. [27]. They appear to be administered to fish without causing any negative side effects, unlike chemotherapeutics. Additionally, they are cost-effective, readily accessible, biocompatible and contribute a significant role in sustainable and rural community development [28] (Table 1).

**Table 1:** List of medicinal plants and their potent bioactive compounds for the possible therapeutic use in various diseases of aquaculture.

Scientific name	Common name	Part used	Bioactive compounds	Properties	References
*Scutellaria baicalensis*	Chinese skullcap	Aerial part	Baicalin, baicalein, 7-*O*-glucuronide and oroxylin A	Antimicrobial, antioxidant, anticancer, and anti-inflammatory	[29]
*Castanea sativa*	Sweet chestnut	Phenolic extract of shell	Trigalloyl-HHDP-glucose, gallic acid and quercetin	Antibacterial and antioxidant	[30]
*Pandanus tectorius*	Screw pine	Leave powder extract	p-hydroxybenzaldehyde, syringaldehyde, E-ferulaldehyde, E-sinapinaldehyde, vanillin and 5-hydroxymethylfurfual	Antibacterial and antioxidant	[31]
*Aloe vera*	Aloe-vera	Leaves	7-hydroxyaloin A and 7-hydroxyaloin B, (8-O-methyl-7-hydroxyaloin A and 8-O-methyl-7-hydroxyaloin B	Antibacterial, antifungal, and antiviral properties	[32]
*Elaeagnus angustifolia*	Russian olive	Leaves extract	Cyanidin-3-O-glucoside, gallic acid and anthocyanin	Antimicrobial, antioxidant and antimutagenic	[33]
*Coffea arabica*	Arabian coffee	Coffee silver skin	Chlorogenic acids, caffeine, trigonelline, melanoidins and diterpenes	Antibacterial	[34]
*Citrus limon*	Lemon	Lemon peels	Caffeoyl N-Tryptophan, hydroxycinnamoyl-Oglucoside acid, vicenin 2, eriocitrin, kaempferol-3-O- rutinoside, and quercetin-3-rutinoside	Antibacterial and antifungal	[35]
*Nigella sativa*	Black cumin	Seed	Thymoquinone, thymohydroquinone, dithymoquinone, p-cymene, carvacrol, 4-terpineol, t-anethole, sesquiterpene, α-pinene, and thymol	Antibacterial	[36]
*Arum maculatum*	Cuckoo pint	Leaves	1,1-diphenyl 2-picrylhydrazyl free radical (DPPH), β-Carotene and tocopherols	Antimicrobial activity, antioxidant properties, antibacterial, antimutagenic, anticarcinogenic and cardioprotective activities	[37]
*Aloe barbadensis*	Aloe vera	Leaf	Aloe-emodin, aloin, aloesin, emodin and acemannan	Antifungal, antibacterial, antiviral and anthelmintic	[38]
*Thymus vulgaris*	Common thyme	Oil	Borneol, carvacrol, cymol, linalool, thymol, tannin, apigenin, luteolin, saponins and triterpenic acid	Antibacterial, antifungal and antioxidant	[39]
*Achillea cucullata*	Gandrain	Oil	Camphor, 1,8-cineole and isoborneol	Antioxidant, antibacterial antimicrobial and enzyme-inhibition activity	[40]
*Anisomeles malabarica*	Malabar catmint	Leaves	β-sitosterol, ovatodiolide, anisomelicacid, malabaric acid, anisomelol and triterpene betulinic acid	Antioxidant, antibacterial and antibacterial activities	[41]
*Cynara cardunculus*	Cardoon	Oil	5-O-caffeoylquinic, 3,5-O-dicaffeoylquinic acid, luteolin-7-O-glucoside, luteolin-7-O-malonylhexoside, palmitic, linoleic, stearic, caproic and oleic acid	Antioxidant, anti- inflammatory, antifungal and antibacterial	[42]
*Melocanna baccifera*	Muli bamboo	Leaf	β-sitosterol, E-phytol, β-amyrin, syringic acid, blumenol B and tianshic acid	Antifungal, antibacterial, antiprotozoal, antibacterial antitussive and immunomodulatory	[43]
*Thymus linearis*	Himalayan thyme	Oil	Thymol, carvacrol, thymyl acetate and β-caryophyllene	Antimicrobial, antibacterial antioxidant activity and antiseptic	[44]
*Excoecaria agallocha*	Mangrove	Leaf	Squalene, tochopherol, terpenoids	Antimicrobial, antibacterial and immunomodulatory	[45]
*Mentha piperita*	Peppermint	Oil	Menthone, iso-menthone, menthol, germacrene D, α-pinene, Limonene, 1,8-cineole and menthone	Antimicrobial, antibacterial and Immunostimulant	[46]
*Ocimum sanctum*	Tulsi	Leaves	Ursolic acid, oleanolic acid and salrigenin	Antioxidative, antimicrobial, antistress, antibacterial antidiabetic and antiviral	[47]
*Citrus medica*	Fingered citron	Fruit	Limonene, geranial and neral	Antifungal and antibacterial	[48]
*Zingiber officinale*	Zinger	Root	Zingiberene, β-bisabolene, α-farnesene, β-sesquiphellandrene, and*α*-curcumene, 6-gingerol and 6-shogaol	Antioxidants, antibacterial, anti-inflammatory and Antimicrobial	[49]
*Cinnamomum cassia*	Chinese cinnamon	Tree bark	Cinnamaldehyde, cinnamon oil, eugenol, salicylaldehyde and *trans*-cinnamic acid	Antioxidant, anti- inflammatory and antibacterial	[50]
*Eriobotrya japonica*	Japanese medlar	Leaves	Corosolic acid, 3-epicorosolic acid, euscaphic acid, oleanolic acid, maslinic acid (9), methyl arjunolate and betulinic acid	Antioxidant, anti- inflammatory and antibacterial	[51]
*Tinospora cardifolia*	Guduchi	Leaves	Berberine, choline, tinosporin, tinocordiside, furanolactone and β-sitosterol	Antibacterial	[52]
*Withania somnifera*	Ashwagandha	Root	Withaniol, withasomnine, somnirol, somnitol, withanic acid, phytosterol and ipuranol	Antibacterial	[53]
*Toona sinensis*	Chinese cedar	Leaves	Ursolic acid, bBetulic acid, cedrellin, phytol and scopoletin	Antibacterial, antiviral, antioxidant, anti-cancer and anti-inflammatory	[54]
*Punica granatum*	Pomegranate	Leaves	Ellagic and gallic tannins	Antiviral and antibacterial	[55]
*Thymus daenensis*	Thyme	Oil	Thymol, p-cymene, 1,8-cineole, γ-terpinene and carvacrol	Antiseptic, antimicrobial, antispasmodic, antibacterial antioxidant and antitussive agent	[56]
*Indigofera suffruticosa*	Indian indigo	Leaves	Syringic acid, p-coumaric acid, vanillin, syringaldehyde, salicylic acid, quercetin, isoliquiritigenin, and formononetin	Antibacterial	[57]
*Camellia sinensis*	Tea plant	Leaves and buds	Catechins, epicatechins, theaflavins, flavonol glycosides, l-theanine, caffeine and theobromine	Antiparasitic and antibacterial	[58]
*Allium sativum*	Garlic	Tuber	Allicin, alliin, diallyl sulfide, diallyl disulfide, diallyl trisulfide, ajoene, and S-allyl-cysteine	Hypolipidemic, antibacterial, antimicrobial, antihypertensive and hepatoprotective	[59]
*Carica papaya*	Pawpaw	Seeds	Tannins, papain, nicotine, cyanogenicglucosides and quercetin	Antioxidative, antibacterial and antimicrobial	[60]

The enhancement and acceleration of the aquaculture sector growth require the development and production of effective, safe and pollution-free herbal compounds. Herbal medicines are inexpensive and have excellent results. Additionally, they are eco-friendly and green [61]. Pharmacology and toxicology of numerous herbal medicines and compound preparations lack functions that treat aquatic animals, prevent disease, promote growth and improve the quality of aquatic products. These abilities are found in effective ingredients, content, structure, extraction and relationships among effective ingredients. Various nations are now actively pursuing new methods of green farming and investing more money in scientific research. As current society develops to environmental protection and healthy direction, aquaculture is also no exception [62]. With the help of a combination of extract chemicals or erstwhile immunostimulants, they can be used as a whole plant or specific part. Being environmentally cheaper, medicinal plants show minimum side effects and are hence used as an option for antibiotics in the fisheries industry. The relevance of plants as natural and undamaging composite has probable in aquaculture as a substitute for antibiotics [63]. The aquaculture sector relies on phytotherapy since they have proven benefits such as improving the delivery system, bioavailability, and sustained discharge of bioactive compounds [64].

## 2 Characters of *Aeromonas hydrophila*

### 2.1 Morphological characters

Features such as capsule formation and motile with flagella formation were observed [65]. Isolates of *A. hydrophila* produce lateral flagella for surface movement/swarming and polar flagella for suspension movement. Polar flagella production in *A. piscicola* AH-3 has been examined with mutations in *flaAB, flaH, fliA, fliM*, *maf*-*1* and *flrC* eliminate polar flagella production and resulting in decreased adhesion and biofilm formation [66]. In addition to having single lateral flagellin, *A. piscicola* AH-3 contains glycosylated polar and lateral flagella. On the other hand, *A. hydrophila* AH-1, has two lateral flagellins but just one glycosylated polar flagellum [67]. In *A. piscicola* AH-3, mutations in the pseudaminic acid biosynthesis genes *pseB* and *pseI* prevented the production of both polar and lateral flagellin, whereas, in *A. hydrophila* AH-1, only the development of polar flagella was impacted. Thus, in glycosylation-negative *A. hydrophila* AH-1 mutants, lateral flagella production was unaffected [68].

### 2.2 Physiological characters

Maximum temperature for growth in nutrient broth (30, 37 and 41 °C); growth factor requirements using a mineral-ammonium medium containing glucose or succinate as the sole source of carbon and energy; growth in peptone water in the presence or absence of sodium chloride; catalase production; growth in KCN medium; methyl red and Voges-Proskauer reactions [69].

### 2.3 Carbohydrate metabolism

Production of acid and gas from glucose and glycerol: acid production from L-arabinose, L-rhamnose, L-xylose, D-mannose, D-cellobiose, D-lactose, D-maltose, D-sucrose, D-trehalose, D-mannitol, D-dulcitol, D-sorbitol, salicin, sorbose, raffinose, erythritol, mucate, adonitol, meso-inositol, melibiose; esculin hydrolysis; production of butanediol-dehydrogenase and *β*-galactosidase [70].

### 2.4 Nitrogenous compound metabolism

Production of urease, lysine decarboxylase, phenylalanine deaminase, tryptophan deaminase, ornithine decarboxylase, arginine dihydrolase, H_2_S production on Kligler's medium and from cysteine on cysteine-iron agar, tetrathionate reductase; indole formation in peptone water [71].

### 2.5 Extracellular enzymes

Biochemical and physiological characteristics of the *A. hydrophila* isolates have been shown in Table 2 [72]. They were found to possess the same characteristics as those tested [73, 74]. Production of elastase, lipase, gelatinase, pectinase, RNAase and DNAase [75].

**Table 2:** The comparative study of characteristics of *A*. *hydrophila* isolates [72].

Characters	Characterization [73]	Characterization [74]
Gram stain	–	–
Shape	Rod	Rod
Motility	+	+
Oxidase	+	+
Catalase	+	+
OF test	F	F
Acid and gas production from glucose	+	+
Acid production from		
Lactose	+	+
Sucrose	+	+
Maltose	+	+
Mannitol	+	+
Inositol	–	–
Sorbitol	–	–
Rhamnose	–	–
Methyl-red test	–	–
Voges-Proskauer	+	+
Indole	+	+
H_2_S production	+	+
Arginine decomposition	+	+
Lysine decarboxylation	–	–
Ornithine decarboxylation	–	–
Citrate utilization	+	+
Growth in 4 °C	–	–
Growth in 5 °C	–	–
Growth in 37 °C	+	+
Growth in 40 °C	+	+
Growth in 0% NaCl	+	+
Growth in 1% NaCl	+	+
Growth in 2% NaCl	+	+
Growth in 3% NaCl	+	+
Growth in 4% NaCl	–	–

### 2.6 Structural proteins, phospholipids and polysaccharides

*O*-antigens, capsules and S-layer proteins serve as protection mechanisms against host defences. Capsules have anti-phagocytic activity, improve resistance to the complement system and promote adhesion in *Aeromonas* sp. [76,77]. *O*-antigens are a type of lipopolysaccharide with a variety of structural properties that act as colonization factors. *A. piscicola* at 20 °C, AH-3 produces *O*-antigens but not at 37 °C, resulting in *O*-antigens-deficient strains that are unable to colonize hosts and have low T3SS component expression [78]. *A. hydrophila* has eight distinct O-antigen gene clusters, and all epidemic strains isolated from channel catfish (*Ictalurus punctatus*) share a homologous O-antigen gene cluster [79]. The S-layer protein gene (ahsA) encodes an exterior paracrystalline layer in *A. hydrophila* TF7 (genomic data lacking). During insertional mutagenesis of spsD S-protein secretion, this layer is removed [80].

## 3 *A. hydrophila* growth in culture media

Rimler Shotts agar was used as a selective medium to isolate *A. hydrophila* (HiMedia). Plates were incubated at 37 °C for 28 h. Using an automated microbial analyzer all cultures were identified to the species level (Biolog, US). For further characterization, selected *A. hydrophila* colonies were subcultured in Tryptic Soya Broth (Difco). In the RS-medium, *A. hydrophila* formed yellow colonies. Gram staining of these colonies gives a Gram-negative reaction, microscopically examination gives rod-shaped, motile colonies, biochemical tests give oxidase-positive, antibiotic and fermentative resistance tests give novobiocin resistance, indicating that the colonies are made up of aeromonads. By using an automated microbial analyzer, all isolates were identified as *A. hydrophila* [81].

Rimler-Shotts (RS) medium was created, which is a modification of various enterobacteria-specific media. It was made up of L-ornithine hydrochloride 0.8 g; L-lysine-hydrochloride 6.5 g; sodium thiosulfate 5 g; agar 13.5 g; maltose 3.5 g; sodium deoxycholate 1.0 g; L-cysteine-hydrochloride 0.3 g; novobiocin 0.005 g; sodium chloride 5.0 g; bromothymol blue 0.03 g; ferric ammonium citrate 6.8 g; yeast extract 3.0 g and sufficient water in quantity to make 1 L. Stirring was used to dissolve the components, the pH was adjusted to 7.0 and the liquid was brought to a boil for 1 min, cooled to 45 °C and poured into plates. Plates were refrigerated until they were required. When organisms were inoculated on RS media, four distinct kinds of colonies were obtained. The first was yellow indicating that maltose fermentation had occurred. The second was yellow with a black center and showed a similar reaction to the first but with H_2_S added. The third sort of colony displayed hues of greenish-yellow to green, indicating lysine or ornithine decarboxylation, or both. The fourth type had a black center and was green, implying the same reaction as the third but with H_2_S production [[82], [83], [84]]. The most basic (maltose fermentation) or acidic reaction was produced by choosing and combining the components in this medium (decarboxylation of lysine or ornithine, or both). Sodium thiosulfate and L-cysteine hydrochloride, or both, were largely required for the production of hydrogen sulphide, with ferric ammonium citrate being used to aid in the visualization of this reaction. Gram-positive organisms and *Vibrio* spp. were eliminated with the addition inhibitors of sodium deoxycholate and novobiocin. The use of novobiocin to suppress the development of *Vibrio* spp. reduces the misunderstanding that can occur when distinguishing these organisms from anaerogenic strains of *A. hydrophila* [85].

Smooth, spherical, small, convex and yellowish colonies of *A. hydrophila* (CAHH14 strain) were seen on the RS-plate. It passes biochemical tests for motility, catalase, oxidase and the O/F test, and it is resistant to the novobiocin and 0/129 disc [86]. All *Aeromonas* strains were cultivated for 24–36 h in Tryptic soy broth (TSB) (Difco) at 28–30 °C. The accuracy tests were conducted using TSA as a control medium, as well as for colony separation from recovery media. In the selectivity experiments, the reference agar used was plate count agar (Difco). For the recovery of *A. hydrophila* from water samples, the following selective media were used. DNTA consisted of 30 mg of ampicillin (Sigma, USA) and 0.1% toluidine blue (Sigma, USA) added to DNase agar (Difco). MacConkey agar (Difco) with 1% trehalose was used as MCT (Difco). *A. hydrophila* AB3-15 was cultivated as a lawn culture in Roux bottles for 24 h on Tryptic soya agar (TSA) and collected in sterile phosphate-buffered saline (PBS) pH 7.0. A live vaccination was created using recently obtained cells, and a dead vaccine was created by heating the harvested cells in a water bath at 60 °C for 1 h (Table 3) [87,88]. Rimler Shotts agar, a selective medium, was used to isolate *A. hydrophila* (HiMedia). The plates were incubated for 48 h at room temperature (RT 28 °C). Using differential biochemical assays, all cultures were identified at the species level. For additional molecular characterization, selected *A. hydrophila* colonies were sub-cultured in peptone water [89].

**Table 3:** The list of selective media of pure culture used in the growth of *A. hydrophila* [87].

Culture medium	Acronym
Pril-xylose-ampicillin modified agar	PXAm
Inositol-brilliant green-bile salts agar	IBB
Bile salts-brilliant green agars	BBG
Rimler-Shotts agar	RS
Rimler-Shotts agar (without lysine)	RSm
mA agar	mA
DNase-toluidine blue-ampicillin agar	DNTA
Xylose-sodium deoxycholate-citrate agar	XDC
Starch-bile salts agar	SB
MacConkey-trehalose agar	McT
Dextrin-fuchsin-sulfite agar	DFS

**Fig. 1:**
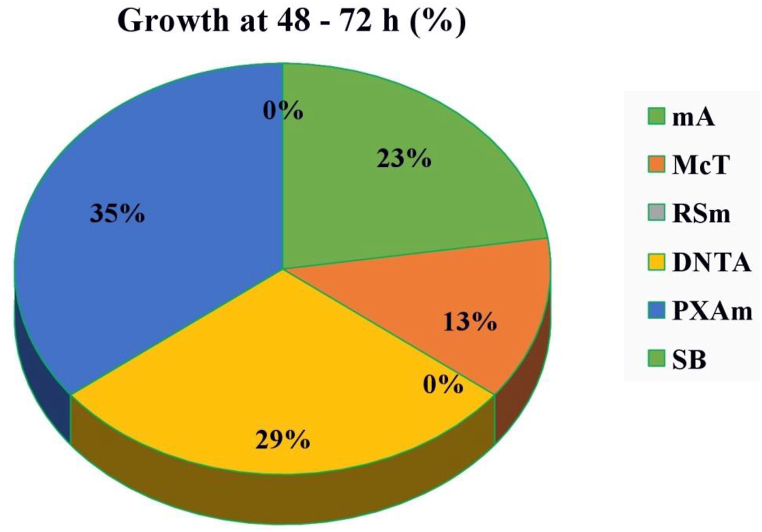
Qualitative growth of *A. hydrophila* on the selective recovery media [90].

**Fig. 2:**
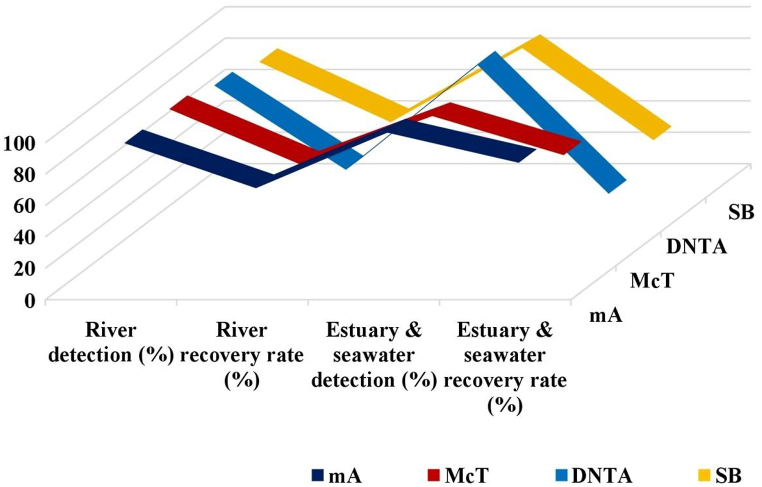
Comparison of the efficiency of different media for recovery of *A. hydrophila* from water samples [90].

The various strains of *A. hydrophila* grew in all 6 media tested. On mA, McT, PXAm and DNTA agars, one strain developed after 48 h. However, due to their ability to significantly reduce the growth of both Gram-positive and Gram-negative flora, these four media were the most selective (other than *Aeromonas* and *Plesiomonas* spp.) (Fig. 1) [90]. mA agar has a high specificity; the percentage of colonies identified as *A. hydrophila* was greater than 75% and only 3% of the colonies on this medium had false-negative results (Fig. 2) [90]. The percentage of non-typical colonies identified as *A. hydrophila* in the other medium ranged from 20 to 33.3% for DNTA and McT agars, respectively, while the percentage of typical colonies that were positively verified ranged from 22.2 to 60% for SB and McT agars, respectively [91].

The total bacterial count was done on Tryptone soya agar (TSA, Oxoid) plates and *Aeromonas*-like bacteria were isolated on AIM plates [92]. Small, convex, round, smooth, translucent and yellow colonies were formed by *A. hydrophila* isolates (Ah1 and Ah12) on both Aeromonas Isolation Agar (AIA) medium and Rimler Shotts (RS) medium. *A. hydrophila* was a small rod with polar flagella that moved swarmingly and was Gram-negative. *A. hydrophila* isolates consume sucrose, lactose, fructose, dextrose, glucose, D-maltose, D-galactose, D-ribose, glycerol, sorbitol, trehalose, starch, rhamnose, L-arginine, salicin, D-mannose, amygdalin and arabinose but do not ferment raffinose; *A. hydrophila* strains do not grow in 4.8% NaCl but do in nutritious broth and other basal media with 0.2% NaCl [65].

## 4 Gross clinical and pathological symptoms

*A. hydrophila* has been identified as the causative agent of many symptoms related to gastroenteritis, systemic infections and bacterial endocarditis in humans and other species. *A. hydrophila* have reportedly been linked to necrotic septicemic and ulcerative disorders in amphibians, reptiles and fishes [93]. It is recognized as an opportunistic pathogen of homeothermic and poikilothermic hosts [89]. *A. hydrophila* causes disease in fish known as Motile Aeromonas Septicemia (MAS), Hemorrhagic septicemia, ulcer disease or red-sore disease [94]. Bacterial infections cause heavy mortality in both wild and cultured freshwater fish. *A. hydrophila*, *A. sobria* and *A. caviae* are the most prevalent *Aeromonas* sp. [95]. According to Taylor [96] *A. hydrophila* and *A. sobria* are the pathogens that cause Motile Aeromonas septicemia (MAS) in fish and other aquatic species. In West Bengal, Karunasagar et al. [97] investigated outbreaks of infectious dropsy brought on by *A. hydrophila* in three of the most common species of Indian major carps. Bacterial fish disease, particularly bacterial hemorrhagic septicemia and Motile Aeromonas Septicemia in freshwater fish resulted in substantial losses [9,98,99]. *Aeromonas* infection was responsible for 45.45% of exotic carp diseases, followed by 6.25% of Indian main carp diseases. *Aeromonas* spp. was previously described in exotic carp, *H. molitrix*, *C. idella* and *C. carpio* [92,100]. Motile *Aeromonas* spp. has been isolated from water, healthy or diseased fish, food products, human feces and other clinical/environmental samples [101]. Fish are stressed when the quality of the water deteriorates, which increases their susceptibility to infections from opportunistic pathogens such as *Aeromonas* species [102,103]. The increasing prevalence of *Aeromonas* in diseased populations of Indian major carps and exotic carps shows that it is evolving into a significant pathogen as the carp culture system is intensified [9].

The majority of freshwater fish affected by *A. hydrophila* are catfish, various species of bass and a variety of tropical ornamental fish*. A. hydrophila* causes infections in fish resulting red mouth, bloated abdomen, blood on the exterior surface and around the anal scale sloughing, surface lesions and septicemia [92,93]. Clinical indications such as loss of balance, abnormal movement, reddish lesions on the fin bases and anal area and a greyish-white lesion that extended up to the caudal fin were observed in each group of intramuscularly injected fish in a moribund state. The liver was found to be enlarged, unsmooth and irregular after the dissection of the freshly dead fish [104]. It is believed to be the cause of fatal hemorrhagic septicemia and epizootic ulcerative syndrome (EUS), which are characterized by internal symptoms like ascetic fluid accumulation, organ damage, anaemia, especially to the kidney and liver as well as external symptoms like blisters, dropsy, abscesses, gill and anal haemorrhages, exophthalmia, scale protrusion, tail rot and fin rot [105,106]. *A. hydrophila* was isolated from dropsy-infected common crap (*C. carpio*) in Meghalaya which caused enormous mortality [86]. *Saprolegnia declina* infection in salmonids, spring viraemia of carp and myxobacterial and other protozoan infections in the larval branchial cavity are only a few of the diseases that *A. hydrophila* makes worse [107]. Fish mortality caused by *A. hydrophila* causes significant economic losses in the Southeast Asian fish farming business [19,108]. The skin abnormalities resembled furunculosis, assuming the shape of very big conspicuous bulges filled with clear exudate that, when ruptured, revealed haemorrhagically altered muscle. The skin lesions started as depigmented patches surrounded by a hyperaemic zone with ulcer formation. Inside the abdominal cavity, some fish had exophthalmos, inflammation around the pectoral fins, hyperaemia of the swim-bladder wall and petechial haemorrhages on the liver. Low erythrocyte counts, low haematocrit and haemoglobin levels were used to identify severe anaemia. Lower levels of total protein, cholesterol, triacylglycerol and total calcium, as well as an increase in urea, were found in clinical chemistry examinations of the diseased fish. Among the enzymes and isoenzymes examined, α-hydroxybutyryl dehydrogenase, lactate dehydrogenase, alanine aminotransferase and γ-glutamyl transferase all reported catalytic concentrations exceeding multiples of the normal range [95]. The symptoms vary due to a variety of factors such as the presence or absence of septicemia, organisms virulence and fish resistance to infection and stress factors linked with the fish. The diagnosis of this disease based solely on symptoms is extremely unreliable and may have devastating financial effects on the fish producer due to the variety of symptoms [94].

## 5 Pathogenicity of *A. hydrophila*

The *A. hydrophila is* advocated as an indicator of the presence of harmful chemicals in surface waters, such as phenol, but this was not supported by further investigations. Another potential application of aeromonads as a water quality indicator is the link between aerogenic and anaerogenic strains [109]. The anaerogenic strains of *A. hydrophila* predominated in sewage-polluted river water, whereas aerogenic strains predominated in unpolluted waters [8,110,111]. *A. hydrophila* outbreaks are generally thought to be associated with changes in host susceptibility caused by environmental changes such as elevated temperature which is connected to the generation of virulence elements including cytotoxins and hemolysins as well as elevated nitrite levels in farmed fish and hypoxic circumstances [112]. The ability of *A. hydrophila* to form biofilms, use particular metabolic pathways and regulate the expression of virulence factors via quorum sensing are all examples of virulence factors. The disease is also brought on by the production and/or secretion of virulence factors like hemolysins, cytotoxins, adhesins, proteases and lipases [113]. According to research endotoxin, haemolysin, enterotoxin and cytotoxin are now known to be produced by aeromonads [8,[114], [115], [116], [117], [118], [119], [120]].

**Fig. 3:**
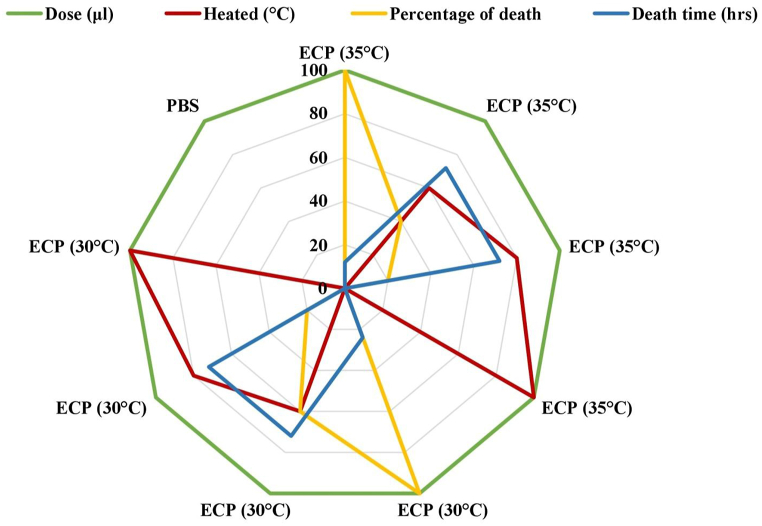
Lethal toxicity of *A. hydrophila* (CAHH14 strain) to rohu [86].

According to Daskalov [15] *A. hydrophila* pathogenicity and virulence are dependent on its ability to produce components related to gastroenteritis. Endotoxins, exotoxins, siderophores, cytotoxins, adhesins, invasins, S-layers and flagella are examples of these properties. Elastase, collagenase, metalloprotease, enolase, lipase and serine protease are all degradative enzymes found in *A. hydrophila* spp. that can contribute to virulence [121]. Slime formation, haemolysin, proteolytic activity, antimicrobial peptides, enterotoxin, lipolytic activity, aerolysin, cytosine and gelatinase have all been identified as virulence factors in *A. hydrophila*. These elements are used by *A. hydrophila* as a mechanism of defence, survival and pathogenicity establishment [12]. The uncontrollable accumulation of bacterial microcolonies on surfaces that are encased in a polysaccharide matrix is known as a biofilm. Bacterial resistance to conventional antibiotics and chronic infections come from biofilm formation [122]. Bacterial resistance to antimicrobial agents and host defences is provided by biofilms [123]. The primary virulence factors that influence pathogenicity are extracellular toxins (hemolysin, enterotoxin and protease), structural traits (pilli, S-layer and lipopolysaccharide), adhesion and invasion [4,124]. In refrigerated conditions, *Aeromonas* species can develop and produce toxins, demonstrating that refrigeration is ineffective in controlling the infection [122]. Studies on the proteolytic activity of ECP of *A. hydrophila* found that the culture grown at 30 °C had the highest proteolytic activity. However, the ECP from the culture grown at 35 °C exhibited only minimal proteolytic activity (Fig. 3) [86]. The proteolytic effect and found that at 28 °C, specific strains of *A. hydrophila* have the least proteolytic effect [125]. This result was solely due to ECP obtained from cultures grown at various temperatures during incubation [86]. *Aeromonas* strain causes fluid accumulation in adult rabbit's ligated ileal loops, similar to *Vibrio cholerae* toxigenic strains [8].

Many environmental conditions like temperature, pH values, salt levels and others influence the production of virulence traits. *A. hydrophila*, which has been associated with human gastroenteritis, is probably capable of growing in foods at refrigeration temperatures currently thought to be sufficient for preventing the growth of food-borne pathogens [15]. Human disease is most likely caused by adhesion to and colonization of mucosa followed by fluid buildup or epithelial alteration [126]. Studies have shown that pathogens produced toxins more quickly at 28 °C and that the inclusion of 1–5% NaCl or a pH of more or less than 7.2 decreased the formation of hemolysin and cytotoxin [121]. In research, it was found that out of 69 strains of *A. hydrophila* about 47 strains produce hemolysin titer at 10 °C while only 6 strains produce hemolysin titer at 37 °C. Regardless of the hemolytic titer, 40% (4 strains) of *A. hydrophila* were enterotoxin after growing at 37 °C, while 30% (3 strains) were enterotoxin after growing at 10 °C [127]. When the bacteria were cultured at 35 °C for 30 h, the largest amount of haemolysin was produced. The bacteria's highest level of proteolytic activity was seen after 36 h of growth at 30 °C [86].

Extracellular Products (ECP) were collected using a modified procedure [128]. Centrifugation at 2800 rpm for 45 min was used to extract the bacterial cells from the culture broth samples. Using the recovered supernatant fluid as a source of crude ECP, the hemolytic and proteolytic activities were assessed [129,130]. The approach was used to determine the hemolytic activity and proteolytic activity of ECP [86]. The rohu was shown to be fatal to the hemolytic and proteolytic toxin generated by *A. hydrophila* (LD_50_ 1.7 × 10^4^ CFU/ml). Heating ECP reduced its lethality while boiling it at 100 °C for 10 min rendering it completely inactive. This shows that the temperature affected the protease and hemolytic activities of *A. hydrophila* ECP [125]. Fish mortality is significantly influenced by the heat-labile potential pathogenic component of ECPs (protease and hemolysin) when fish are injected with untreated ECP of *A. hydrophila* (CAHH14 strain) [131].

## 6 Antibiotic resistance pattern

**Fig. 4:**
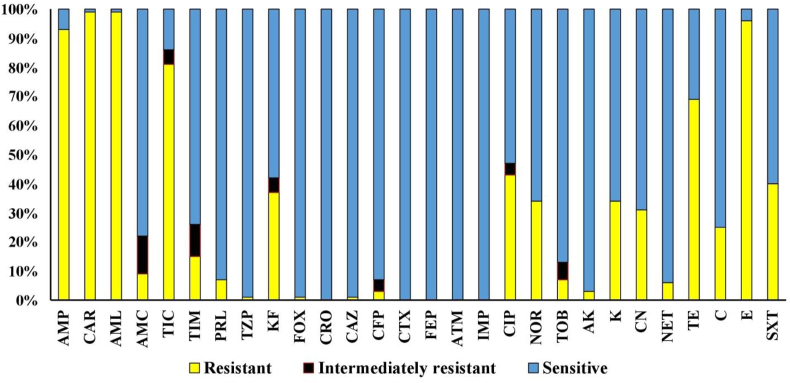
Susceptibility profile (%) to antibiotics of *A. hydrophila* (n = 67) isolates [7].

The *A. hydrophila* isolates were sensitive to sulfamethoxazole, streptomycin, chloramphenicol, neomycin and trimethoprim/sulfamethoxazole [132]. Ampicillin, bacitracin, penicillin, tetracycline and streptomycin were all resistant, whereas erythromycin, gentamycin, kanamycin, nalidixic acid, neomycin and sulfisoxazole were all susceptible. Oxytetracycline, chlortetracycline, tetracycline, neomycin, trimethoprim/sulfamethoxazole and chloramphenicol were all effective against the majority of the isolates [15]. Multiple antibiotic resistances were found in 319 strains of *A. hydrophila* isolated from fish and prawns [133]. Methicillin and rifampicin resistance was the most common, followed by bacitracin and novobiocin resistance, although chloramphenicol sensitivity was the most common [15]. The *A. hydrophila* strains was sensitive to azithromycin, ofloxacin, oxytetracycline, doxycycline, streptomycin, chlortetracycline, nitrofurazone and norfloxacin but resistant to ampicillin, amoxicillin, bacitracin, cloxacillin, cefuroxime, co-trimoxazole, cephalexin, erythromycin and flumequine [65]. It was found except *Aeromonas diversa*, 96% of the *Aeromonas* spp. tested sensitive for ciprofloxacin (Fig. 4) [7,134]. They found that 63% of the 90 *Aeromonas* strains isolated from freshwater fish were susceptible to ciprofloxacin. In order to control the bacterial population in India's fields and hatcheries, a variety of antimicrobial drugs (oxytetracycline, ciprofloxacin, nitrofurantoin, furazolidone or chloramphenicol) were used [135].

The *A. hydrophila* isolates were resistant to quinolones, aminoglycosides, fluoroquinolones, cephalosporins of the third and fourth generations and other frequently used antibiotics but susceptible to cephalosporins (ceftazidime, cefuroxime, cefpodoxime, ceftriaxone, cefalotin, cefoxitin, cefotaxime and cephalexin), norfloxacin, nitrofurantoin, quinolones, chloramphenicol, tetracycline, kanamycin, aminoglycosides, amoxicillin, sulphamethoxazole, imipenem, streptomycin, oxytetracycline, doxycycline, gentamicin, ticarcillin, ofloxacin, pefloxacin, ciprofloxacin, neomycin, oxacillin, gatifloxacin, amikacin and levofloxacin identified in several global environmental samples, clinical samples and diseased fish samples [65]. Oxysentin, acimox and oxy-D Vet had the lowest, medium and greatest inhibitory zones respectively. The sensitivity of the bacterium *A. hydrophila* to oxysentin, acimox and oxy-D Vet was determined to be low, medium and extremely sensitive respectively. A prescribed combination of acimox and oxy-D Vet may be used to cure anal erosion. Caudal fin ray loss, ulcerative lesions and hemorrhagic lesions all fully recovered [104]. In Taiwanese isolates of *Aeromonas* discovered growing resistance to trimethoprim, sulphamethoxazole, tetracyclines, certainly extended-spectrum cephalosporins (ceftriaxone, cefotaxime and cefotaxime) and tobramycin [136]. Cotrimoxazole often works well against *Aeromonas* species despite neither sulphamethoxazole nor trimethoprim being very powerful against these bacteria, because the two medications work well together [137]. The *Aeromonas* species (*A. hydrophila*, *A. caviae* and *A. sobria*) found in this investigation were all sensitive to ofloxacin, pefloxacin and ciprofloxacin based on the antibiotic profile. All three species (*A. caviae, A. sobria* and *A. hydrophila*) were tetracycline, nitrofurantoin and augmentin resistant, whereas ceftriaxone, gentamycin, cotrimoxazole and amoxicillin were randomly sensitive. Resistance to oxytetracycline is common in environmental *Aeromonas* isolates [138]. The majority of the isolates in the study were resistant to first-generation quinolones (oxolinic acid and pipemidic acid) but clinically responsive to fluoroquinolones ranging from pefloxacin (54% to ciprofloxacin 98%) [136]. This study supports *Aeromonas* sp. sensitivity to ciprofloxacin, pefloxacin and ofloxacin. All of the *Aeromonas* species identified in this investigation were completely sensitive to these antibiotics. The frequency and character of antibiotic resistance differed depending on the source of the strains [139]. *Aeromonas* spp. resistance to frequently used antibiotics is a growing issue in ornamental fish. Previously, it was discovered that the species *Aeromonas* was becoming more resistant to β-lactam antibiotics [140,141].

Ampicillin, carbenicillin, amoxicillin, cephalothin and cefoxitin were the least effective β-lactam antibiotics for *A. hydrophila*, whereas amikacin was the most effective aminoglycoside antibiotic (84%). Furthermore, *A. hydrophila* exhibited resistance to quinolones (ciprofloxacin and norfloxacin) of around 40% [7]. Compared to other species, *A. hydrophila* isolates had greater rates of ciprofloxacin and norfloxacin resistance (43% and 34%, respectively). Quinolones are artificial antibiotics that are frequently used as the initial line of treatment for human infections with *Aeromonas* [137,142]. Fourteen antibacterial agents from 9 different antibiotic groups including cephalosporins, aminoglycosides, tetracyclines, chloramphenicol, nitrofurantoin, fluoroquinolones, sulphonamides, penicillin and polymixin were used to test the 8 isolates of *A. hydrophila* found in 15 samples of fish taken from retail stores in Mhow city that were tested in-vitro. Cefuroxime, ciprofloxacin, ceftriaxone, cefotaxime, gentamycin, chloramphenicol, nalidixic acid, kanamycin, nitrofurantoin and ofloxacin had the highest sensitivity (100%) followed by co-trimoxazole (62.2%) and oxytetracycline (50%). Antibiotics ampicillin and colistin were both resistant to all of the isolates, that means none of the isolates tested positive for penicillin or the polymyxin group of antibiotics. All *A. hydrophila* isolates were positive for multiple drug resistance [143].

## 7 Experimental induction of *A. hydrophila* infection

The intramuscular injection technique resulted in 100% death at a dosage of 2.8 × 10^6^ CFU/fish and 60% mortality at a dose of 2.8 × 10^5^ CFU/fish of the experimental fish. *H. molitrix* was found to be sensitive to *A. hydrophila* as evidenced by 100% mortality at 2.8 × 10^6^ CFU/fish and 60% mortality at 2.8 × 10^5^ CFU/fish. The post-infection mortality days ranged from 2 to 5 days and 4 to 9 days respectively. Through experimental infections in carps (rohu, catla and mrigal) 100% of *L. rohita* died at a dose of 6.7 × 10^6^ CFU/fish and 80% died at a dose of 6.7 × 10^5^ CFU/fish, with post-infection mortality days ranging from 1 to 4 days and 3 to 11 days respectively [144]. *Cirrhinus cirrhosus* mortality was 100% at a dosage of 6.7 × 10^6^ CFU/fish and 60% at a dose of 6.7 × 10^5^ CFU/fish, with post-infection mortality days ranging from 2 to 5 days and 4 to 12 days, respectively [104].

The pathogenicity of *A. hydrophila*, a bacterial isolate found in naturally diseased singhi fish (*Heteropneustes fossilis*) was tested for pathogenicity against catfishes (*H. fossilis* and *C. batrachus*), carps (*L. rohita, C. catla* and *C. cirrhosus*) and perch (*A. testudineus*) with average body weights of 20.4 g for *H. fossilis*, 25.6 g for *C. batrachus*, 35.2 g for *L. rohita,* 25.7 g for *C. catla*, 30.5 g for *C. cirrhosus* and 20.3 g for *A. testudineus*. Intramuscular injections of 6.7 × 10^6^ and 6.7 × 10^5^ CFU/fish were performed. Pathogenicity of injected *A. hydrophila* was confirmed at 30 °C water temperature by the death of 60 to 100% of all examined fishes within 2 to 11 days. All of the examined fishes had *A. hydrophila* infections in their livers, kidneys and intestines. According to studies, the bacterial load in catfish livers ranged from 5.5 × 10^8^ CFU/g in *H. fossilis* to 5.6 × 10^7^ CFU/g in the intestines of *C. batrachus*. The liver of *C. batrachus* and the kidney of *H. fossilis* were found to have the lowest bacterial load 2.4 × 10^3^ CFU/g and 2.2 × 10^2^ CFU/g respectively. The liver of *C. catla* had 4.9 × 10^9^ CFU/g, the intestine of *L. rohita* had 7.7 × 10^8^ CFU/g, and the intestine of *C. cirrhosus* had 5.8 × 10^8^ CFU/g of bacteria respectively. In the kidneys of *C. catla*, *L. rohita* and *C. cirrhosus*, the lowest bacterial loads were 2.7 × 10^4^ CFU/g, 3.3 × 10^4^ CFU/g and 5.6 × 10^3^ CFU/g respectively [144].

Twelve fish from each group were exposed to the pathogenic *A. hydrophila* strain 018 after a 60-day feeding period (obtained from Aquatic Animal Health Management Division, CIFE, Mumbai). The *A. hydrophila* was cultured in a BOD incubator for 24 h on nutrient broth at 30 °C before being collected by centrifuging the culture broth at 10,000 rpm for 10 min at 4 °C. The final concentration was maintained at 1.8 × 10^8^ CFU/ml by serial dilution after the cells had been washed three times in sterile PBS (pH 7.4). Each experimental group fish received an intraperitoneal injection of 0.2 ml of bacterial solution. For ten days, all groups were monitored for mortality. *A. hydrophila* was found to be the cause of mortality when tissues from dead fish were obtained for bacteriological culture. Fish exposed to *A. hydrophila* after the challenge had damaged hepatocytes, oedema and leucocytic infiltration in parenchymatous tissues, haemosiderosis and acute bleeding in the kidney [88]. *Ictalurus punctatus*, a channel catfish, was exposed to the pathogen *A. hydrophila* by abrading its skin and submerging it in a suspension of the pathogen, which caused a lesion to form. Lesions developed that were comparable to those brought on by intraperitoneal (IP) injection [145]. Injecting common infections such as *A. hydrophila*, *Aquaspirillum* sp., *Pseudomonas* sp., *Streptococcus* sp. and *Streptococcus* sp. into healthy *C. batrachus* and *Ophiocephalus striatus* resulted in mild, mild-to-moderate and severe dermo-muscular necrotic lesions [146]. *Anguilla anguilla* eels were exposed to extracellular products isolated from *A. hydrophila* and *A. jandaei*, which had LD_50_ values of 10^7^ and 10^8^ CFU/fish respectively and caused degenerative changes and ulceration [147].

## 8 Isolation and calculation of *A. hydrophila*

The *A. hydrophila* was isolated from Thai pangus, it has the bacterial load of 4.8 × 10^6^ to 7.2 × 10^7^ CFU/g in the gut, 2.6 × 10^6^ CFU/g in the liver and 2.4 × 10^3^ to 3.70 × 10^6^ CFU/g in the kidney [113]. *A. hydrophila* was isolated from *H. fossilis* [72]; they found that the liver had the greatest bacterial load 2.4 × 10^7^ CFU/g, while the kidney had the lowest 2.1 × 10^2^ CFU/g. The total bacterial load detected in the sampled fish gut, liver and kidney were 1.0 × 10^5^ to 1.5 × 10^5^ CFU/g, 2.7 × 10^2^ to 4.5 × 10^4^ CFU/g and 1.0 × 10^3^ to 2.2 × 10^3^ CFU/g, respectively [92]. Moribund fish liver, kidney and intestine were homogenized and sterile PS was used to make two consecutive decimal dilutions of 10^−1^ and 10^−2^ from the stock solution for each organ. To assess the pathogens pathogenesis in the organs of the experimentally infected fish, the colonies that developed were counted using a computerized colony counter [148]. *H. fossilis* kidney had the lowest bacterial load 2.1 × 10^2^ CFU/g, while the *H. fossilis* liver had the highest 2.42 × 10^7^ CFU/g bacterial load. When singhi fish were experimentally infected with the selected *A. hydrophila* isolate (CK602), 100% of the fish died within 1 to 9 days at a dosage of 1.92 × l0^7^ CFU/fish [72]. A haemolysin-negative mutant of *A. hydrophila* was used to immunize fingerlings of *C. catla*, *L. rohita* and *C. mrigala*. The highest antibody titers were found in *C. catla* followed by *C. mrigala* and *L. rohita*. Fish that had received an immunization showed good resistance to homologous challenges. When faced with heterologous challenges *C. mrigala* and *L. rohita* displayed a moderate level of resistance [97].

## 9 Prevention and control of *A. hydrophila*

Disinfectants and antimicrobial medicines have shown minimal effectiveness in the prevention or control of aquatic animal diseases. One of the most revolutionary technologies that have emerged in response to these challenges is the use of “immunostimulants” which fill the gap left by vaccines and probiotics [149]. The best way to avoid *A. hydrophila* infection is to never have it. This may sound absurd, but fish are considerably less susceptible to this disease if stress factors such as handling, stocking levels, diet, transportation and water quality are minimized. To limit the possibility of this disease arising, excellent cleanliness and filtration processes are essential. Treatment should begin as soon as the diagnosis of *A. hydrophila* infection in fish is established [94]. Using antibiotics to prevent disease and promote growth may lead to the emergence of drug-resistant microorganisms and the buildup of antibiotic residues in fish and the environment [150,151]. Furthermore, chemotherapy has the potential to destroy or disrupt the natural bacteria in the digestive tract, which is helpful to fish [152].

The positive benefits of some beneficial bacteria in aquaculture have been widely established [[153], [154], [155]], these helpful bacteria are referred to as probiotic bacteria. Probiotic bacteria are being used to manage possible infections, which is an alternate technique that is gaining popularity in the aquaculture industry [156]. Probiotics are microorganisms that enhance the host health. They are used in aquaculture to control disease and as supplementary nutrients [157]. To develop a vaccine for trout as well as a detection kit for food-borne diseases caused by *A. hydrophila* in Korea, the isolation and characterization of *A. hydrophila* in Korea is required as the initial step. Diverse techniques including PCR, biochemical/physiological assays, randomly amplified polymorphic DNA (RAPD), plasmid profiling and gel electrophoresis of total membrane and extracellular proteins were used to characterize and compare different strains of *A. hydrophila* to the type strain. Hemolysin, haemagglutinin, cytotoxin, protease and surface array proteins were among the virulence factors [158]. A study indicates that when the fingerlings were intra-peritoneally challenged with *A. hydrophila* there was a rise in TLC in the control (infected) group, but the levamisole-supplemented groups had a decrease in leucocyte count. This is mostly due to the fish immune systems reaction to the bacterial invasion. The gradual restoration of leucocyte counts to normal in the immunostimulant-supplemented groups may be indicative of the repair of systemic injury [159].

Antimicrobial peptides or proteins (AMPs) are the first line of defence molecules occurring naturally in all multicellular species. AMPs have a significant role in innate host defence in nature. Because they include all of the major AMP types including cathelicidins, hepcidins, defensins, histone-derived peptides and piscidines, fish is regarded as a notable source of antimicrobial peptides. AMPs are thought to be a very promising all-natural antibiotic replacement. The fish peptides have broad-spectrum antibacterial activity, eliminating infections that affect both fish and humans. Additionally, their genes are highly reactive against microorganisms and innate immunostimulatory chemicals and they have immunomodulatory properties. Later studies have shown that several of the unique characteristics of fish peptides such as their capacity to function even in conditions of extremely high salt concentrations, make them promising candidates for development as therapeutic antimicrobials. Numerous biological effects of AMPs have been seen, including the neutralization of endotoxin, immunomodulatory action and stimulation of angiogenesis [160].

When fish were challenged intraperitoneally with *A. hydrophila* the total serum protein concentration was lowest in the control (infected) group and highest in the D3 group at the end of the experimental trial [161]. Gudding et al. [162] found that total serum protein content was considerably increased in levan-fed common carp fingerlings against *A. hydrophila* infection, whereas lower values were reported in the control (infected) group. Two antibiotics Remet-30®, a potentiated sulfonamide and Terramycin®, oxytetracycline are the only ones now approved for use in therapy. According to Swann and White [94] terramycin®, oxytetracycline and Remet-30®, a potentiated sulfonamide is the only antibiotics on the market right now (Table 4).

**Table 4:** The recommended doses of useful drugs currently used in aquaculture.

Product	Sponsor	Dosage
Terramycin®	Pfizer, Inc.	2.5–3.75 g/100 lb of fish per day for 10 days in a feed
Remet-30®	Hoffman-La Roche, Inc.	50 mg/kg of fish per day for 5 days

Another approach to using antibiotics is a dip or bath. However, the efficacy or effectiveness of this strategy is debatable. This method has drawbacks such as destroying indoor tank systems biofilters and perhaps preventing the uptake of antibiotics into the fish. Inadequate dose levels, overdose, bacterial drug resistance and the chelation of calcium to hard water in the case of Terramycin® used in a dip or bath are all potential problems with antibiotic therapy. Remember that many fish may be stressed even if they show no symptoms of this disease, and the increased handling required for therapy could be fatal for these species [94]. Fish vaccines have the potential to significantly reduce certain disease-related losses, therefore reducing antibiotic use. As a result, overall unit costs are reduced, and manufacturing is more predictable. Fish vaccines are preferable to antibiotics because they are made of natural biological materials that do not leave a residue on the product or the environment and do not result in the development of a disease-causing organism that is resistant to them; however, there are some drawbacks, such as the decreased value of the cultivated species and the slowed growth rate of some species [20,162].

Multiple injections of β-glucan produced from barley might improve immune response and disease resistance in *L. rohita* fingerlings against infections caused by opportunistic pathogens *A. hydrophila* and *E. tarda*. β-glucans are glucose polymers present in the plant, fungus and bacterium cell walls that have been shown to have immunostimulatory activities in fish [163]. Because they are comparable to fungal or bacterial Gram-negative polysaccharides, fish recognize these polysaccharides as foreign agents. Following exposure, the immune system of fish develops an inflammatory response similar to that of a disease, providing excellent protection against opportunistic infections [164]. Numerous investigations have found that β-glucan increases fish resistance to a variety of bacterial infections by increasing complement and lysozyme levels, as well as improving the phagocytic, respiratory burst and bactericidal activities of fish phagocytes. By administering various doses of β-glucan four times every two weeks, as was shown in the fourth week following the challenge both by I.P. injection and bath immersion, the mortality (%) due to infections with *A. hydrophila* and *E. tarda* was significantly reduced (*P* < 0.05). The group of fish that received 10 mg of β-glucan/kg of body weight four times had the lowest mortality (%) rate [163].

### 9.1 Probiotics

The possibility of inhibiting two *A. hydrophila* strains by bacteriocin-producing lactic acid bacteria isolated from retail slices of beef [165]. According to Vescovo et al. [166] *A. hydrophila* survival in ready-to-use mixed salad greens may be hampered by combinations of carbon dioxide, *Lactobacillus casei* and low storage temperature. According to Santos et al. [167] and Daskalov et al. [15] *Lactococcus lactis* sub-sp. Lactis strain 388 displayed inhibitory effects against three strains of *A. hydrophila*.

### 9.2 Polyphosphates/NaCl

According to Palumbo et al. [168] *A. hydrophila* in BHI broth was shown to be inactivated by a mixture of 2% of any polyphosphate (sodium pyrophosphate, sodium tripolyphosphate and hexaphos or sodaphos) and 3.5% NaCl and this inactivation was temperature-dependent. The polyphosphate NaCl mixture inhibited bacterial growth in ground pork during refrigerated storage. According to Velazquez et al. [169] the growth of *A. hydrophila* was completely inhibited by concentrations between 0.5 and 3.0% of four phosphates (tetrasodium pyrophosphate, sodium acid pyrophosphate, trisodium phosphate and sodium tripolyphosphate) in modified completely defined synthetic medium (mCDS) and cooked ground meat medium. A stronger inhibitory impact (bactericidal and bacteriolytic effects) was produced by sodium acid pyrophosphate (0.5%) [15].

### 9.3 Heating

D-values (1.5, 0.10 and 0.03 min) were obtained at 51, 57 and 60 °C indicating that such heat methods can provide a significant safety factor in the inactivation of *A. hydrophila* in liquid egg [15,170].

### 9.4 High hydrostatic pressure

The *A. hydrophila* response to high hydrostatic pressure, which ranged from 51 to 304 MegaPascals (MPa), was examined for 15 min. The results showed *A. hydrophila* has the potential to repair or proliferate after being exposed to pressure in pork [171].

### 9.5 Smoking

According to Boyle et al. [172] several *A. hydrophila* strains were sensitive to the concentration of smoke from various types of wood smoke. Fish is traditionally preserved by the cold-smoking method.

## 10 Control of *A. hydrophila* by herbal treatment in aquaculture

Antibiotics should not be used routinely during fish culture to reduce disease risk because they may harm the indigenous microflora of juveniles or adults and may increase the possibility of developing antibiotic-resistant bacteria [173]. As a result, eco-friendly disease-prevention methods are needed to support long-term fish culture. Immunostimulants, which improve fish resistance by increasing non-specific defence mechanisms are an intriguing option for disease management. Immunostimulants are a class of biological or synthetic compounds that, when used as an adjuvant with a vaccination, stimulate non-specific defence mechanisms as well as specific immune responses [174]. Although food modification is an excellent method for improving non-specific immunity in fish, research has been conducted to examine the impact of dietary variables on the immune system [88]. The use of herbs to prevent *A. hydrophila* ulcerative dermatitis, either through dip therapy or by adding herbs into feeds, is another innovative alternative approach to the control of aquaculture disease [175,176]. According to Hao et al. [177] plant extracts (eugenol and pimento extracts) were shown to be the most efficient in suppressing *A. hydrophila* growth [15,20].

In vivo testing must be carried out to examine the immunomodulatory and disease resistance impacts of plant materials to assess their full potential on fish health and disease resistance. One of the most critical elements in determining the effectiveness and safety of phytotherapy is dosage, which is directly related to the material used. While too low of a dosage may not have the intended impact on fish, too high of a dosage may be toxic and have detrimental consequences on fish development, survival and immunological function [178,179]. One of the factors considered essential to the effectiveness of the experiment is the time duration of treatment. Choosing the ideal treatment time is crucial for obtaining numerous benefits. To maximize the impact of plant-enriched diets on fish immunity and disease resistance, several researchers have focused on finding the ideal treatment time. On the other hand, environmentally friendly ingredients should be recommended (powdered plants or extracts with low-toxicity solvents). According to the plant and the type of material used, the dose of the plant provides an essential characteristic that must be evaluated carefully [[180], [181], [182]] (Table 5).

**Table 5:** Disease resistance of various medicinal plants against various fish pathogens used in aquaculture.

Medicinal plants	Common name	Against fish	Pathogen	Disease resistance	References
*Bougainvillea glabra*	Paper flower	*Cyprinus carpio*	*Aeromonas hydrophila*	yes	[183]
*Allium hirtifolium*	Mooseer	*Oncorhynchus mykiss*	*Streptococcus iniae*	yes	[184]
*Andrographis paniculata*	King of bitters	*Pangasianodon hypopthalmus*	*Aeromonas hydrophila*	yes	[185]
*Eichhornia crassipes*	Water hyacinth	*Oncorhynchus mykiss*	*Streptococcus iniae*	yes	[186]
*Ginkgo biloba*	Maidenhair trees	*Cyprinus carpio*	*Aeromonas hydrophila*	yes	[187]
*Sargassum angustifolium*	Narrow leaf weed	*Oncorhynchus mykiss*	*Yersinia rukeri*	Yes	[188]
*Aloe barbadensis*	Aloe vera	*Oncorhynchus mykiss*	*Saprolegnia parasitica*	Yes	[38]
*Melocanna baccifera*	Muli bamboo	*Labeo rohita*	*Saprolegnia parasitica*	Yes	[43]
*Thymus vulgaris*	Common thyme	*Cyprinus carpio*	*Saprolegnia* spp.	Yes	[189]
*Phyllanthus niruri*	stonebreaker	*Oreochromis mossambicus*	*Vibrio harveyi*	Yes	[190]
*Urtica dioic*a	Stinging nettle	*Labeo victorianus*	*Aeromonas hydrophila*	Yes	[182]
*Ocimum sanctum*	Tulsi	*Labeo rohita*	*Aeromonas hydrophilia*	Yes	[47]
*Euphorbia hirta*	Asthma-plant	*Cyprinus carpio*	*Aeromonas hydrophila*	Yes	[191]
*Thymus vulgaris*	Common thyme	*Oreochromis mossambicus*	*Streptococcus iniae*	Yes	[192]
*Sophora flavescens*	Kushen	*Oreochromis niloticus* (GIFT)	*Streptococcus agalactiae*	Yes	[193]
*Cynodon dactylon*	Bermuda grass	*Catla catla*	*Aeromonas hydrophilia*	Yes	[194]
*Lactuca indica*	Indian lettuce	*Epinephelus bruneus*	*Streptococcus iniae*	Yes	[195]
*Withania somnifera*	Ashwagandha	*Labeo rohita*	*Aeromonas hydrophila*	Yes	[53]
*Zingiber officinale*	Ginger	*Oncorhynchus mykiss*	*Aeromonas hydrophila*	Yes	[196]
*Aegle marmelos*	Golden apple	*Oreochromis mossambicus*	*Vibrio harveyi*	Yes	[197]
*Solanum trilobatum*	Purple fruited pea eggplant	*Oreochromis mossambicus*	*Aeromonas hydrophilia*	Yes	[198]
*Piper longum*	Indian long pepper	*Epinephalus tauvina*	*Vibrio harveyi*	Yes	[199]
*Echinacea purpurea*	Purple coneflower	*Oreochromis niloticus*	*Pseudomonas fluorescens*	Yes	[200]
*Tinospora cordifolia*	Guduchi	*Oreochromis mossambicus*	*Aeromonas hydrophila*	No	[201]
*Allium sativum*	Garlic	*Oreochromis niloticus*	*Aeromonas hydrophila*	Yes	[202]

Neem, *Azadirachta indica* is an Indian plant that has been researched extensively across the world. In India, neem is known as “Sarva roga nivarak” or “healer of all ailments” and is considered an important part of the Ayurvedic tradition. According to research, the water-soluble portion of the alcoholic extract of *A. indica* leaves showed hypoglycemic, hypolipidemic, hepatoprotective, antifertility, hypotensive and anti-serotonin activities. Neem *A. indica* tree oil possesses antibacterial properties that are effective against a variety of Gram-positive and Gram-negative bacteria, including strains of *Mycobacterium* TB and streptomycin-resistant bacteria [203]. Some of the bioactive substances that give neem its antibacterial effects include azadirachtin, nimbidinin, nimbinin, nimbidic acid, nimbidin, nimbin, nimbolide, margolone, isomargolonone, margolonone, tetra-notriterpenoids and limnoids [204,205]. The most well-known biopesticide is *A. indica*, which has been categorized by WHO/UNEP as a naturally occurring pesticide with “high” environmental effects. Myxobolasis, trichodinosis, gyrodactylosis, argulosis, scuticocliates and other parasite diseases in farmed tropical freshwater fish have all been treated with natural remedies including plant extracts [206]. Azadirachtin is beneficial against *Argulus* spp. [207]. and its influence on physiological and serum biochemical markers in *Carassius auratus* has also been studied [208]. Fish treated with azadirachtin exhibited significantly higher TEC, TLC, total Ig, total protein, NBT activity, serum lysozyme activity and myeloperoxidase levels than the control group (*P* < 0.05) in all treatment groups. Similar results were obtained for SGOT, SGPT and blood glucose levels, however, PCV and Hb did not differ substantially (*P* < 0.05) between the treatment and control groups. Azadirachtin exhibited considerably (*P* < 0.05) improved relative percentage survival (42.60%) against *A. hydrophila* infection at a dosage of 4 g/kg compared to the control. Azadirachtin EC 25% (4 g/kg) was shown to have increased serum lysozyme, NBT activity, leucocyte counts, protein profiles and resistance to *A. hydrophila* infection in this study, suggesting that it might be used as an immunostimulant in aquaculture [149].

Many synthetic and herbal immunostimulants have been found to improve fish immunological health by increasing phagocytic, lysozyme and complement activities as well as immunoglobulin levels in response to several causative extremities [209]. Traditional medicine has used a variety of plants to treat and control several diseases [210]. According to reports, natural plant products with active principal components like flavonoids, alkaloids, pigments, terpenoids, steroids, essential oils and phenolics have appetite-stimulating, anti-stress, growth-promoting, tonic immunostimulatory and anti-microbial properties in finfish and shrimp larviculture [149,211]. The growing interest in the use of herbal immunostimulants to improve fish defence systems and protect them from diseases is relatively new. There are many different types of herbal plants, but the *Ocimum sanctum* (Tulsi) is regarded as the “Queen of Herbs” and its medicinal benefits are well-documented in Hindu mythology. The bioactive principle in *O. sanctum* leaf extracts such as ursolic acid, oleanolic acid and saligenin have immunomodulatory properties [212]. Eugenol, methyl eugenol and caryophyllene are among the several components found in tulsi *O. sanctum* leaves in addition to water-soluble phenolic compounds [47,213]. After being exposed to *A. hydrophila*, the control group displayed significantly damaged hepatocytes, oedema and leucocytic infiltration in parenchymatous tissues, as well as severe bleeding and haemosiderosis in the kidney. In contrast, the T5 group supplemented with 1.25% levan only experienced mild renal tubule deterioration. The T5 group shows the highest relative survival percentage of juveniles after being challenged with *A. hydrophila* followed by the T4 group [88].

The plant species that have shown the greatest promise for usage in the aquaculture industry are garlic (*Allium sativum*), ginger (*Zingiber officinale*), pomegranate (*Punica granatum*), Bermuda grass (*Cynodon dactylon*) and ashwagandha (*Withania somnifera*). Allicin and ajoene, two components of pure garlic have been found to have an impact on aquaculture and to be effective against harmful microorganisms (*A. hydrophila*, fish protozoa *Spironucleus vortens* and *Ichthyophthirius multifiliis*) by stimulating the immune system [214]. Pomegranates contain a variety of phytochemicals, such as the bioactive polyphenol ellagitannins, which have antioxidant and anti-inflammatory properties. The chemical makeup of *C. dactylon* includes tannins (catechins), phenolic substances (gallic acid), flavonoids (quercetin) and anthocyanins (cyanidin). Bermuda grass (*C. dactylon*) has antiviral, antiparasitic, immunostimulant, antibacterial and growth-regulating properties in fish and shellfish [180,215,216]. Various characteristics of *W. somnifera* include antiviral, antibacterial, growth-promoting effects and immunostimulant [53]. Along with certain sesquiterpenoids and zingiberene as the primary component, ginger is made up of a mixture of zingerone, shogaols and gingerols (Table 6) [250].

**Table 6:** List of medicinal plant species wherein plant part used, their doses and experimental conditions developed through the exposure of various treatments and optimization strategies.

Medicinal plant species	Common name	Part used	Doses	Experimental conditions	Fish used	References
*Asparagus racemosus*	Shatavari	Root extract	100 mg/kg	FRP tanks	*Labeo rohita*	[217]
*Achyranthes aspera*	Prickly chaff flower	Leaf and seed powder	0.5%	Aquarium	*Clarias batrachus*	[218]
*Azadirachta indica*	Neem	Leaf extracts	5, 7 and 10%	Tanks	*Oncorhynchus mykiss*	[219]
*Allium sativum*	Garlic	Root powder	0.5, 1.5, 3 and 10%	Fibre pond	*Lates calcarifer*	[220]
*Nymphaea alba*	Water lily	Whole plant	10, 20 and 30%	Plastic aquaria	*Clarias gariepinus*	[221]
*Phyllanthus emblica*	Amla	Fruit extract	20 mg/kg	Tanks	*Oreochromis* *niloticus*	[222]
*Thymus vulgaris*	Thyme	Flower oil	2%	Fiberglass tank	*Oncorhynchus mykiss*	[223]
*Zingiber officinale*	Ginger	Rhizomes of ginger	5 g, 10 g, 15 g, and 20 g/kg	Semi-intensive culture system	*Labeo rohita*	[224]
*Artemisia absinthium*	Common wormwood	Aqueous extract	0.5, 1 and 1.5%	Concrete tank	*Cyprinus carpio*	[225]
*Coffea arabica*	Coffee	Coffee silver skin	10, 20, 40 and 80 g/kg	Biofloc system	*Oreochromis niloticus*	[34]
*Psidium guajava*	Guava	Leaves extracts	100, 150, 200 and 250 leaves mg/kg	Plastic tanks	*Cyprinus carpio*	[226]
*Nigella sativa*	Black seed	Seeds powder	1 and 2.5%	Glass aquaria	*Labeo rohita*	[36]
*Achyranthes aspera*	Prickly chaff flower	Leaf and seed powder	0.25, 0.5% leaves and 0.5% seeds	Hapa (pond)	*Labeo rohita*	[227]
*Isatis tinctoria*	Woad	Leaves extract	1, 1.5, 2 & 2.5%	Tanks	*Pseudotropheus acei*	[228]
*Radix bupleuri*	Chaihu	Root extract	200–800 mg/kg	Cage	*Epinephelus lanceolatus* and *Epinephelus fuscoguttatus*	[229]
*Ziziphus jujube*	Fruit extract	Fruit extract	0.25, 0.5 and 1%	Tank	*Cyprinus carpio*	[230]
*Citrus sinensis*	Common jujube	Peels	5, 10 and 29 g/kg	Biofloc system	*Oreochromis niloticus*	[231]
*Curcuma longa*	Turmeric	Root powder	2.0 g/kg	Fiberglass tank	*Cyprinus carpio*	[232]
*Mespilus germanica*	Spanish cherry	Leaves extract	0.25, 0.5 and 1%	Fiberglass tank	*Cyprinus carpio*	[233]
*Urtica dioica*	Stinging nettle	Plant methanolic extracts	0.1 and 0.5 g/kg	Aquarium	*Oncorhynchus mykiss*	[234]
*Musa acuminata*	Banana	Peel flour	0, 1, 3, 5 and 7%	Plastic tank	*Labeo rohita*	[235]
*Chlorophytum borivilianum*	Safed musli	Root (Polysaccharide)	0.4%	Plastic quarantine tank	*Labeo rohita*	[236]
*Ocimum sanctum*	Holy basil or Tulsi	Leaf extracts	0.05, 0.1, 0.2, 0.5 and 1%	Rectangular plastic tub	*Labeo rohita*	[47]
*Mentha piperita*	Peppermint	Leaves extract	1, 2, 3, 4 and 5 g/kg	Round blue tanks	*Lates calcarifer*	[237]
*Euphorbia hirta*	Hairy spurge	Leaf extract	5, 10, 20, 25 and 50 g/kg	Tanks	*Cyprinus carpio*	[191]
*Nigella sativa*	Black cumin	Seed oil	1, 2, 3%	Aquarium	*Oncorhynchus mykiss*	[238]
*Sophora flavescens*	Chinese medicinal herb	Root powder	0.025, 0.05, 0.1, 0.2 and 0.4%	Concrete tanks	*Oreochromis niloticus*	[193]
*Viscum album*	Mistletoe	Leaf extract	50 mg/kg	FRP tank	*Oreochromis* *niloticus*	[239]
*Lindera aggregate*	Chinese spicebush	Root	60 mg/l	Glass aquarium	*Carassius auratus*	[50]
*Pseudolarix kaempferi*	Golden larch	Leaf extract	12.5 and 25 mg/ml	Glass petri dishes	*Carassius auratus* eggs	[240]
*Eriobotrya japonica*	Japanese plum	Leaf extract	0.1, 1 and 2%	Recirculating cement tanks	*Epinephelus bruneus*	[241]
*Prunella vulgaris*	Carpenter's herb	Ethanol extract	0.01, 0.1 and 1%	Recirculating cement tanks	*Paralichthys olivaceus*	[242]
*Aegle marmelos*	Bel	Leaf extract	5 g/kg	Tanks	*Cyprinus carpio*	[243]
*Datura metel*	Dhatura	Leaf extract	100 mg/ml	FRP tank	Ornamental fish	[244]
*Withania somnifera*	Ashwagandha	Root powder	2 g/kg	FRP tank	*Labeo rohita*	[53]
*Camellia sinensis*	Green tea	Leaf powder	0.5 g/kg	Fiberglass tank	*Oreochromis* *niloticus*	[245]
*Rheum officinale*	Indian rhubarb	Anthraquinone extract (Bail)	0.05, 0.1, 0.2, & 0.4%	Concrete tanks	*Macrobrachium rosenbergii*	[246]
*Lonicera japonica*	Japanese honeysuckle	Plant extract	0.1% and 0.1% with 0.05% boron	Recirculation system	*Oreochromis niloticus*	[247]
*Eclipta alba*	Bhringraj	Leaves aqueous extract	0.01, 0.1 and 1%	FRP tanks	*Oreochromis mossambicus*	[248]
*Cinnamomum cassia*	Cinnamon	Bark extract	75.8–189.6 μg/ml	FRP tank	*Cyprinus carpio*	[249]

## 11 Other plants and perspectives

Some algae and some mushrooms have also been researched for their competency in aquaculture because they are considered a rich source of bioactive molecules; the vast majority of algae showed high antibacterial properties and some showed immunostimulant, antiparasitic, antiviral and antifungal properties [251]. Red alga *Asparagopsis taxiformis* famous to secrete a variety of halogenated metabolites, showed antifungal, antibacterial and antiparasitic properties against several fish pathogens [252,253]. *A. taxiformis* improved the immune system of *Penaeus monodon* and were successful in the therapeutics of vibriosis in *P. monodon,* they found fascinating properties of different marine organisms such as sponges, which can repress quorum sensing of marine pathogenic bacteria such as *Vibrio harveyi*, it represents that they can be used as the better option of medication for the organic aquaculture (Table 7). [[254], [255], [256]].

**Table 7:** List of unused quality medicinal plants in aquaculture.

Scientific name	Common name
*Swertia chiraita*	Chiraita
*Gymnema sylvestre*	Gudmar
*Commiphora wightii*	Guggul
*Lawsennia iermis*	Henna/Mehdi
*Plumbago zeylanica*	Swet chitrak
*Plumbago indica*	Rakta chitrak
*Terminalia chebula*	Harida
*Andrographis paniculata Fam*	Kalmegh/Bhui neem
*Saraca asoca*	Ashok
*Solanum nigrum*	Makoi
*Santalum album*	Sandal wood
*Casia augustifolia*	Senna
*Terminalia bellerica*	Bahada

## 12 Conclusions

Infectious diseases like Motile Aeromonas Septicemia (MAS) are a primary obstacle to the development and sustainability of the aquaculture industry because they cause economic harm, limit productivity and require the use of control measures that are often very expensive. However, overuse of antibiotics and other synthetic pharmaceuticals leads to the development of antibiotic-resistant strains and the accumulation of drug residues in fish tissues and water which could be hazardous to the environment and unsafe to consumers. While effective vaccine development for the number of fish pathogens is usually an expensive and time-consuming process. In addition to vaccination and conventional medications, due to the presence of potent bioactive compounds medicinal plant-derived products appear to be a promising tool for enhancing growth, survival, health status, innate and immune responses, as well as disease resistance in aquaculture. They appear to be administered to fish without causing any negative side effects, unlike chemotherapeutics. Additionally, they are inexpensive, easily available and biocompatible.

Further investigation is strongly recommended to conduct additional research to determine the ideal administration doses and timings as well as to isolate, characterize and quantify the bioactive compounds found in plants and phytoextracts to determine the most potent compounds/metabolites that could be used in new natural formulations for use in fish. Additionally, studies on their mechanism of action, the stability of plant components in aquatic environments, the digestibility in fish, as well as in vitro and in vivo toxicity testing are necessary for their safe utilization. This review article shows the efficacy of phytotherapy in aquaculture which will benefit fish farmers, researchers and pharmaceutical firms.

## Author contribution statement

Anurag Semwal: Conceived and designed the experiments; Analyzed and interpreted the data; Contributed reagents, materials, analysis tools or data; Wrote the paper.

Avdhesh Kumar: Analyzed and interpreted the data; Contributed reagents, materials, analysis tools or data; Wrote the paper.

Neelesh Kumar: Conceived and designed the experiments; Analyzed and interpreted the data; Contributed reagents, materials, analysis tools or data; Wrote the paper.

## Funding statement

This research did not receive any specific grant from funding agencies in the public, commercial, or not-for-profit sectors.

## Data availability statement

Data included in article/supplementary material/referenced in article.

## Additional information

No additional information is available for this paper.

## Declaration of interest's statement

The authors declare no conflict of interest.
